# Pparγ2 Is a Key Driver of Longevity in the Mouse

**DOI:** 10.1371/journal.pgen.1000752

**Published:** 2009-12-04

**Authors:** Carmen Argmann, Radu Dobrin, Sami Heikkinen, Aurélie Auburtin, Laurent Pouilly, Terrie-Anne Cock, Hana Koutnikova, Jun Zhu, Eric E. Schadt, Johan Auwerx

**Affiliations:** 1 Institut de Génétique et de Biologie Moléculaire et Cellulaire, CNRS/INSERM/Université Louis Pasteur, Illkirch, France; 2 Rosetta Inpharmatics, Seattle, Washington, United States of America; 3 A. I. Virtanen Institute for Molecular Sciences, University of Kuopio, Kuopio, Finland; 4 Institut Clinique de la Souris, Illkirch, France; 5 Ecole polytechnique Fédérale de Lausanne, Lausanne, Switzerland; Stanford University School of Medicine, United States of America

## Abstract

Aging involves a progressive physiological remodeling that is controlled by both genetic and environmental factors. Many of these factors impact also on white adipose tissue (WAT), which has been shown to be a determinant of lifespan. Interrogating a transcriptional network for predicted causal regulatory interactions in a collection of mouse WAT from F2 crosses with a seed set of 60 known longevity genes, we identified a novel transcriptional subnetwork of 742 genes which represent thus-far-unknown longevity genes. Within this subnetwork, one gene was *Pparg* (*Nr1c3*), an adipose-enriched nuclear receptor previously not associated with longevity. In silico, both the PPAR signaling pathway and the transcriptional signature of Pparγ agonist rosiglitazone overlapped with the longevity subnetwork, while in vivo, lowered expression of *Pparg* reduced lifespan in both the lipodystrophic *Pparg1/2*-hypomorphic and the *Pparg2*-deficient mice. These results establish Pparγ2 as one of the determinants of longevity and suggest that lifespan may be rather determined by a purposeful genetic program than a random process.

## Author Summary

The progression of aging is controlled by both genetic and environmental factors. Many of these factors are present also in adipose tissue, which itself has been shown to determine lifespan. Applying advanced bioinformatics methods on a large mouse gene expression data set, we identified *Pparg* (*Nr1c3*), an important metabolic controller that regulates the expression of many other genes particularly in adipose tissue, to be associated with longevity. This association was verified in experimental mouse models where the lowered expression of *Pparg* reduced lifespan. In addition to *Pparg*, our analysis identified >700 potential novel aging genes in mouse adipose tissue. More generally, these findings suggest that lifespan may not be a random process but controlled by a purposeful genetic program.

## Introduction

Aging is not a disease, but a natural evolution characterized by declining biological function, whose timeline is sensitive to both environmental and genetic factors. Several longevity candidate genes have been identified, including the insulin/IGF1 signaling pathway [1]–[3]. With the use of dietary regimens, such as caloric restriction (CR) and by modulating core body temperature, the control of energy metabolism has been implicated as a critical determinant of the aging phenotype [4]–[6]. A central physiological component of energy metabolism, involved in energy preservation, is the white adipose tissue (WAT), which has also been directly associated with the determination of lifespan [7],[8]. However, it is still uncertain whether WAT modulates aging via its ability to e.g. store fat, sensitize towards insulin, or produce adipocyte hormones. Also unknown is the nature of the involved genetic players and importantly, whether they function in a purposeful program or as random genetic events.

Using a systems approach we identified a novel subnetwork of genes in mouse WAT, which potentially impacts longevity, suggesting that aging is the result of a determined transcriptional network program and not entirely accidental. Furthermore, the most significantly enriched biological pathway revealed within this aging subnetwork was the PPAR signaling pathway. The aging subnetwork also contained the nuclear receptor *Pparg* (*Nr1c3*), a transcription factor well associated with adipocyte biology [9],[10], but whose contribution to longevity has not been previously assessed. In this study, we support our network theory of aging by demonstrating a significantly altered lifespan in 2 independent genetic mouse models expressing reduced levels of *Pparg*. Thus, in addition to providing novel candidate ‘longevity genes’ such as *Pparg2*, this study also provides further insight into the potential role of WAT biology and genetics as determinants of lifespan.

## Results/Discussion

**Figure 1:**
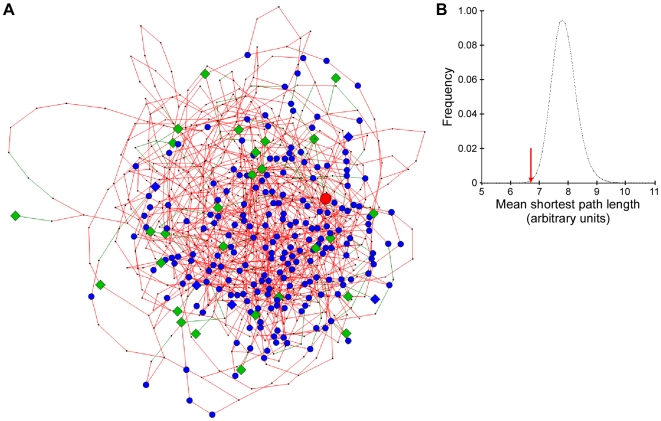
A subnetwork of likely longevity genes in mouse adipose tissue. (A) Longevity-related subnetwork of 775 genes, extracted from the mouse adipose transcriptional network of 13,088 genes. The 33 “known” longevity genes used as a seed set are depicted as green diamonds, and the 213 genes overlapping from the mouse WAT rosiglitazone signature in blue circles. The 5 gene overlap of “known” longevity genes and rosiglitazone signature is shown as blue diamonds. *Pparg*, shown as a red circle, is part of the rosiglitazone signature. (B) The distribution of mean shortest path lengths (μ) for the set of 33 “known” longevity genes and 10^6^ randomly selected sets of 33 genes within the mouse consensus network. Red arrow marks the mean shortest path (μ = 6.7102) for the “known” longevity genes.

We hypothesized that the age-dependent physiological remodeling that leads to phenotypic aging is caused by concerted changes in a longevity-determining genetic network rather than by random changes at the level of individual genes. This hypothesis was tested using a mouse transcriptional network that consists of a union of 4 individual Bayesian networks of predicted causal regulatory interactions in the WAT generated from individual F2 crosses. We interrogated this network of 13088 genes with a seed set of 60 genes, derived from public resources, which either increase or reduce lifespan when genetically perturbed in the mouse (Table S1). Out of these 60 ‘known’ longevity genes, 33 were also present within the adipose tissue network (Table S1; Figure 1A). The pair-wise shortest path analysis against 10^6^ randomly selected sets of 33 genes showed that these 33 genes on average were much more tightly connected than expected by chance (*p* = 0.00149) (Figure 1B). Furthermore, the distribution of the shortest paths within the set of 33 ‘known’ longevity genes was significantly tighter than that for the randomly selected sets as >99% of all Kolmogorov-Smirnov two-sided test *p*-values were less than 0.05. This tight, non-random interconnection of known aging-linked genes suggests that the associated biological phenomena are deliberate such that other ‘unknown’ age-related genes and/or biological processes may be predicted. This network theory is reminiscent of the transcriptional consequences of single genetic perturbations, such as knock-out mouse models or DNA polymorphisms, which result in concentrations of transcriptional changes in the genes functionally relating to the perturbed gene rather than altering genes diffusely distributed across the whole network [11]. Following the concept of using the ‘known’ to discover the ‘unknown’, we thus expanded the subnetwork beyond the 33 longevity genes to other genes most highly connected to them, and obtained a larger subnetwork, containing 742 genes (Table 1, Table S2). By assigning importance to the closeness of connection with known longevity genes, we were thus able to suggest several hundred additional genes that may influence the aging process. One such example, among the top 20 genes for the closeness of connectivity with the 33 ‘known’ longevity genes (Table 1), was the eukaryotic translation initiation factor 4E (eIF4E) binding protein 1 (*Eif4ebp1*, or *4E-BP1*) which, in the unphosphorylated state, represses mRNA translation by binding to eIF4E. Since it regulates adipogenesis and metabolism [12], and one of the mediators of its phosphorylation is insulin signaling [13], Eif4ebp1 can be linked to the established effects of insulin signaling on longevity. Moreover, in Drosophila 4E-BP plays an important role in lifespan extension upon dietary restriction [14]. Eif4ebp1 has furthermore been identified as a “funnel factor” in cancer, through which several oncogenic pathways converge [15].

**Table 1:** Top 20 genes most highly connected to the set of 33 “known” longevity genes in male mouse adipose tissue.

Gene symbol	Rank^a^	Distance^b^	Gene name
Hoxa7	1	4.636	homeo box A7
Npr3	2	4.697	natriuretic peptide receptor 3
Tmem182	3	4.697	transmembrane protein 182
Plxnb2	5	4.758	plexin B2
Fads3	6	4.788	fatty acid desaturase 3
Mcam	7	4.788	melanoma cell adhesion molecule
Mmd	8	4.818	monocyte to macrophage differentiation-associated
1110006G14Rik	9	4.818	RIKEN cDNA 1110006G14 gene
Palmd	10	4.848	palmdelphin
Fry	11	4.879	furry homolog (Drosophila)
Apcdd1	12	4.879	adenomatosis polyposis coli down-regulated 1
Gpt1	13	4.909	glutamic pyruvic transaminase 1, soluble
Prelp	14	4.909	proline arginine-rich end leucine-rich repeat
Eif4ebp1	15	4.909	eukaryotic translation initiation factor 4E binding protein 1
MMG00345348	17	4.909	NA
Echdc3	18	4.909	enoyl Coenzyme A hydratase domain containing 3
Fzd4	19	4.970	frizzled homolog 4 (Drosophila)
Apol6	20	4.970	apolipoprotein L, 6
Rtn2	21	4.970	reticulon 2 (Z-band associated protein)
Smoc1	23	4.970	SPARC related modular calcium binding 1

a Rank within the whole male mouse adipose tissue network of 10,388 genes.

b Distance to the subnetwork of 33 “known” longevity genes.

Biological pathway enrichment analysis is a powerful tool to uncover functional associations within an a priori selected set of genes. When applied to the aging subnetwork of 742 genes (excluding the 33 ‘known’ longevity genes from the full set of 775 genes to eliminate bias), significant enrichment was revealed in several ontology classes with established links to aging such as complement and coagulation cascade (i.e. inflammation), insulin signaling, and ubiquinone pathway (i.e. oxidative stress) (Table 2). Importantly, however, several pathways lacking previously demonstrated association with longevity also appeared among the significantly enriched ontologies. One of these, the PPAR signaling pathway, was actually ranked the highest for the enrichment of all potential longevity genes. Although direct in vivo evidence linking Ppars to aging are scarce, conceptual evidence does exist [16],[17], including links to age-related changes in inflammatory response, insulin sensitivity, distribution and proportion of body fat, oxidative stress [18], and fatty acid oxidation rate. Of the three actual Ppar family members, the only one that was present within the aging subnetwork was Pparγ (Table S2). Given that signaling through Pparγ is also of vital importance to proper adipose tissue development and function [9], [10], [19]–[21], and that Pparγ is regulated in WAT by one of the best established longevity determinants, mammalian *SIR2* orthologue sirtuin 1 (Sirt1) [22], we hypothesized that perturbing Pparγ signaling might affect longevity.

**Table 2:** Pathway analysis of the predicted novel longevity genes in male mouse adipose tissue.

Pathway	Gene count		*p*	Overlap genes
	Overlap	Pathway		
PPAR signaling pathway	11	69	0.00066	Apoa5, Apoc3, Aqp7, Cpt2, Fabp3, Gyk, Pck1, Pparg, Rxrg, Slc27a2, Sorbs1
Ubiquinone biosynthesis	4	9	0.00069	Coq5, MMG00237617, ND5, Ndufa12
Valine, leucine and isoleucine degradation	8	40	0.00078	Acat1, Aldh6a1, Auh, Dld, Echs1, Hibadh, Mccc1, Mcee
Pentose phosphate pathway	6	25	0.00133	Aldob, Fbp1, Fbp2, H6pd, Pgm2, Tkt
Complement and coagulation cascades	10	65	0.00153	C1r, C2, F2, Fgb, Fgg, Hc, Kng1, Plg, Serpine1, Serpinf2
ECM-receptor interaction	11	80	0.00230	Cd44, Col2a1, Col3a1, Col4a1, Col5a3, Fn1, Fndc1, Itga6, Itga7, Spp1, Thbs2
Propanoate metabolism	6	29	0.00300	Acacb, Acat1, Aldh6a1, Echs1, Ldhc, Mcee
Carbon fixation	5	23	0.00536	Aldob, Fbp1, Fbp2, Gpt1, Tkt
Insulin signaling pathway	14	133	0.00760	Acacb, Eif4ebp1, Fasn, Fbp1, Fbp2, Pck1, Pde3b, Pik3r5, Ppargc1a, Ppp1r3b, Pygb, Slc2a4, Socs2, Sorbs1
Glycolysis/Gluconeogenesis	7	51	0.01434	Aldh1a3, Aldob, Dld, Fbp1, Fbp2, Ldhc, Pgm2
Pyruvate metabolism	6	40	0.01514	Acacb, Acat1, Dld, Ldhc, Pck1, Pcx
Alanine and aspartate metabolism	5	32	0.02206	Adssl1, Asns, Dld, Gpt1, Pcx
Fatty acid biosynthesis	2	6	0.03434	Acacb, Fasn
Cysteine metabolism	3	15	0.03845	Cars2, Cdo1, Ldhc
Citrate cycle (TCA cycle)	4	26	0.04157	Dld, Idh1, Pck1, Pcx

The input set of 197 genes was determined by the overlap of the full set of 742 potentially novel longevity genes and the set of 3835 genes for which functional data was available in the KEGG repository at the time of analysis. Note that the 33 “known” longevity genes were excluded from the determination of the input set to remove bias. Only those pathways with *p*<0.05 are shown.

We first tested this hypothesis in silico by using the WAT gene expression signature generated from mice with chemically modulated Pparγ activity through the administration of the Pparγ agonist, rosiglitazone [11]. Notably, 213 out of the 1669 genes whose transcriptional expression was altered by Pparγ activation, overlapped with the genes in the aging subnetwork at a very high significance level (*p* = 5.2028*10^−30^) (Table S2). This finding thus validates the association of *Pparg* with the aging subnetwork and further implicates it as a potential determinant of the aging phenotype.

**Figure 2:**
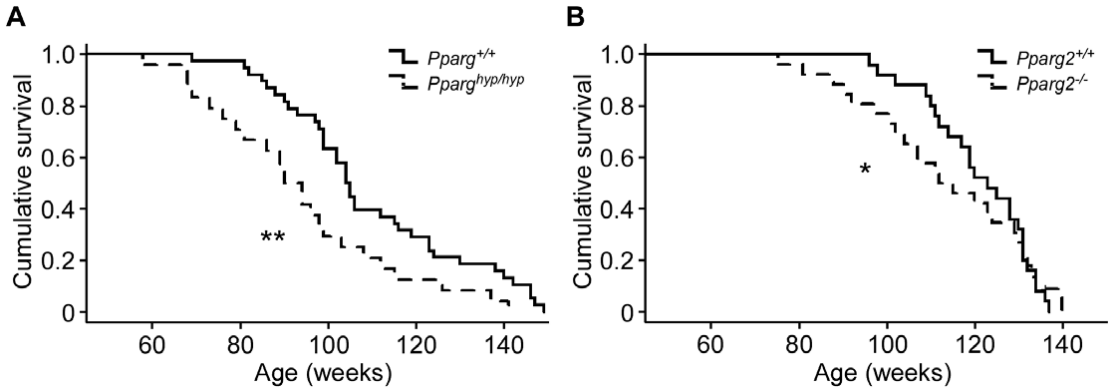
*Pparg* determines longevity. (A) Lifespan of hypomorphic (*hyp*) *Pparg* deficient mice (*n* = 38 wild type and 24 Pparγ^hyp/hyp^ mice). ** *p* = 0.003. (B) Lifespan of *Pparg2* knock-out mice (*n* = 25 wild type and 26 *Pparg2^−/−^* mice). * *p* = 0.020 when mice >120 weeks were excluded from the test.

To put this hypothesis to further rigorous in vivo testing, we investigated the role of *Pparg* in longevity in two mouse models with genetically altered levels of *Pparg* expression: the hypomorphic *Pparg1/2* knock-out mouse, which lacks *Pparg* exclusively in WAT (Figure S1A) and is severely lipodystrophic and remains insulin resistant throughout life [19]; and the *Pparg2* deficient mouse that lacks *Pparγ2* in all tissues (Figure S1B) and shows some features of moderate lipodystrophy and insulin resistance at a young age [23], but which fully compensates upon aging (see below). The nearly complete knockdown of *Pparg1* and *Pparg2* in the WAT of male *Pparg^hyp/hyp^* mice resulted in a reduction in lifespan by approximately 16 weeks when compared to the wild type mice (93.7±4.4 vs 109.6±3.4 weeks, p = 0.03) (Figure 2A). In some respects this observation goes against the prediction that reduced fat mass, as seen during CR [4],[5], would increase longevity; however, if the known insulin sensitizing effects of Pparγ were key to mediating the effects of CR, then one would expect reduced longevity in the *Pparg^hyp/hyp^* mice, where whole body insulin resistance is prominent. However, one potentially confounding factor in this experiment is the profound lipodystrophy exhibited by the *Pparg^hyp/hyp^* mice, which may not represent ‘normal’ metabolic environment due to the amount of metabolic compensation by the upregulation of other signaling pathways that these mice need for survival [19]. Also, although differences in the amount of gross tumors were not observed upon macroscopic necropsy, we can not exclude the possible contribution of more discrete tumors to the decreased longevity of the *Pparg^hyp/hyp^* mice. Interestingly though, the males of an equally lipodystrophic A-ZIP/F-1 mouse model have more than 40% mortality rate before 30 weeks of age [8], in comparison to the *Pparg^hyp/hyp^* mice which survived 85% of the average ∼2 year lifespan of wild type mice. In this sense, *Pparg^hyp/hyp^* mouse model is one of the longest living severely lipodystrophic models reported.

In order to assess more directly the effects of *Pparg* on longevity, without the added complication of reduced adiposity or insulin sensitivity, we made use of *Pparg2^−/−^* mice that we generated in the laboratory and which lack Pparγ2, the WAT enriched Pparγ isoform, in all tissues. Although young *Pparg2^−/−^* mice are lean [23], our ∼2 year old *Pparg2^−/−^* mice had the same total and lean body mass, body fat content (Figure S2A and S2B), and caloric intake (12.33±1.53 vs. 14.24±1.53 kcal/day/mouse, *p* = 0.421) as their age-matched littermate controls. Young *Pparg2^−/−^* mice have also been reported to be insulin resistant [23]. Again in contrast, there were no differences in glucose tolerance, the HOMA index for insulin resistance, nor in circulating insulin or adiponectin levels between our *Pparg2^−/−^* and *Pparg2^+/+^* mice at ∼2 years of age (Figure S2C, S2D, S2E, S2F). Thus, our aging *Pparg2^−/−^* mice represent a very metabolically ‘clean’ model for investigating the role of *Pparg2* in longevity.

Consistent with reduced longevity in the *Pparg^hyp/hyp^* mouse, we noted a significant decrease in lifespan in *Pparg2^−/−^* mice. The female *Pparg2^−/−^* mice lived, on average, 8.8 weeks less than their wild type controls (p = 0.02 when limiting the analysis to those living no more than 120 weeks), although this difference seemed to disappear towards extreme age (Figure 2B). Gross morphological differences that could contribute to mortality were not observed between the genotype groups, although again the contribution of more discrete tumors can not be excluded. Since the *Pparg2^−/−^* mice had reduced longevity, comparable to that in *Pparg^hyp/hyp^* mice, but were not lipodystrophic or insulin resistant, our observations point more towards a specific role for Pparγ2 and any of its downstream pathways in the regulation of longevity, rather than mere changes in fat content and/or insulin signaling. Together our studies thus reveal another genetic factor, *Pparg2*, that affects the basic mechanisms of aging, independent of changes in fat mass or insulin sensitivity [1],[2],[7]. Interestingly, a potential molecular mechanism linking aging and Pparγ has recently been suggested to involve a steroid receptor coactivator-1 (SRC-1) as the age-induced loss of PPARγ/SRC-1 interactions increased the binding of PPARγ to the promoter of a model adipogenic gene for fatty acid binding protein 4 (FABP4, also called aP2) [24].

Both our in silico and in vivo results in the mouse tie longevity tightly together with signaling through Pparγ, and especially the Pparγ2 isoform. We have recently shown increased longevity in knock-in mice carrying the *Ala12* allele of the common human genetic variant *Pro12Ala* variant of *PPARG2* [25], which associates with leanness and improved insulin sensitivity in both man and mouse [25]–[27]. The species gap between mice and humans for the role of Pparγ2 in longevity is bridged by the observation that lifespan is increased also in human carriers of the *Ala12* allele of the *Pro12Ala* variant of *PPARG2* [28]. In the clinical setting, therefore, the links we show between longevity and both *Pparg* and the rosiglitazone signature suggest that thiazolidinediones [29] (TZDs), like rosiglitazone or pioglitazone which are widely used Pparγ agonists and insulin sensitizers in the treatment of type 2 diabetes mellitus (T2D), could be beneficial for longevity. On the face of it, this may in fact seem paradoxical, considering that impaired insulin signaling through insulin receptor or its substrates increases, rather than decreases lifespan in a number of mouse models [1],[2],[7]. However, this can be reconciled by the fact that these models are primarily protected from the detrimental effects of age-induced increase in plasma insulin levels as TZDs lower circulating insulin levels [30],[31]. Fittingly, low insulin levels and maintained insulin sensitivity characterize human centenarians [32]. In light of the above, the results from ongoing outcome trials evaluating the long-term health benefits of treatments with PPARγ-agonists, i.e. TZDs, are eagerly awaited.

In summary, we have identified a substantial set of potential novel longevity genes in mouse adipose tissue, and demonstrate, as a case study, the significant effects of perturbed Pparγ activity on mouse lifespan. Furthermore, our network analysis suggests that, at least in the context of adipose tissue, the determination of longevity may not be a random process, but governed by a concerted effort of a distinct subnetwork of genetic players.

## Materials and Methods

### Ethics statement

Animal experiments were approved by the local ethics committee and performed according to governmental guidelines.

### Compilation of the seed set of 60 “known” longevity genes

To obtain a list of genes with known association to longevity, we used the Phenotypes section of the Mouse Genome Informatics (MGI) resource of The Jackson Laboratory (http://www.informatics.jax.org/) [33], the GenAge Model Organisms pages for mouse within The Human Ageing Genomic Resources (HAGR) [34], and a literature search. The list was compiled in October, 2007.

### Generation of the transcriptional network for mouse adipose tissue

Detailed description of these methods is given in Text S1. In summary, we obtained male adipose tissue gene expression data from 4 different mouse F2 crosses [35],[36] using Agilent microarrays, and generated a Bayesian network for each cross by integrating genetic and gene expression data [37]–[39]. The combined network, containing 13088 nodes and 22809 edges, was obtained as the union of all these 4 separate Bayesian networks.

### Connectivity of “known” longevity genes within the adipose transcriptional network

To assess the degree of connectivity of the 33 ‘known’ longevity genes that were present in the adipose consensus network, mean shortest paths were computed using Dijkstra's algorithm [40] for our set of 33 nodes ( = genes) as well as 10^6^ randomly selected sets of 33 nodes. Briefly, the algorithm finds the smallest number of edges we have to “walk” in order to “travel” from a source node ( = gene) to another node ( = gene) of interest within the map/network. The probability of finding random sets of 33 nodes with shorter mean paths than with our set was obtained by counting the number of such eventualities within the randomized sets, and amounted to a *p*-value of 0.00149, demonstrating that indeed our 33 genes are much more connected within the adipose tissue consensus network than expected by change. Kolmogorov-Smirnov (KS) test was used to further assess whether there were any significant differences between the shortest path distribution within our longevity gene-set and those within each of the 10^6^ random sets. The resulting *p*-value distribution demonstrated that indeed the longevity genes shortest path distribution is not a normal occurrence in the network.

### Generation of *Pparg^hyp/hyp^* and *Pparg2^−/−^* mice

*Pparg2^−/−^* mice were generated from *Pparg^hyp/hyp^* [19] mice by successive matings with transgenic C57Bl/6J mice expressing FLP and Cre recombinases to remove the *Pparg2* specific exon B. All mice studied were backcrossed a minimum of 9 generations to achieve an essentially pure C57Bl/6J background.

### Survival

The original survival cohorts consisted of 38 wild type and 24 *Pparg^hyp/hyp^* male, and 25 wild type and 26 *Pparg2^−/−^* female mice which were maintained on a 12 hour light/dark cycle, fed regular chow, had free access to H_2_O and received standard animal care. The mice were bred locally and were entered into the survival cohort over the course of 23 weeks for male *Pparg^hyp/hyp^* mice, amd 19 months for female *Pparg2^−/−^* mice. For all groups, deaths were recorded weekly. Mice observed as moribund were euthanized and recorded as dead on that week. All *Pparg^hyp/hyp^* reached the end-point, but a few *Pparg2^−/−^* mice survived at the time of analysis.

### Metabolic exploration of *Pparg2^−/−^* mice

Approximately 2 year old wild type (*n* = 5) and *Pparg2^−/−^* (*n* = 9) mice were subjected to the following analysis according to standardized Eumorphia/EMPReSS (http://empress.har.mrc.ac.uk/) protocols: body composition by quantitative nuclear magnetic resonance on a Minispec analyzer (Bruker Optics, The Woodlands, TX), food intake, intraperitoneal glucose tolerance test (IPGTT), and fasting plasma insulin and adiponectin measurements using Ultrasensitive Mouse Insulin ELISA kit (Mercodia, Uppsala, Sweden) and Quantikine Mouse Adiponectin/Acrp30 Immunoassay (R&D systems Inc., Minneapolis, MN), respectively. HOMA index for insulin resistance was calculated from fasting glucose and insulin values [41].

### RNA analysis

*Pparg1* and *Pparg2* gene expression in WAT, BAT, liver and skeletal muscle of *Pparg^hyp/hyp^* mice was previously reported [19] and is presented for comparative purposes. For *Pparg2^−/−^* mice, total RNA was extracted from WAT, BAT, liver and skeletal muscle either with RNeasy for Lipid Tissues Mini Kit (Qiagen, Valencia, CA) or Trizol (Invitrogen, Carlsbad, CA), and reverse transcribed to cDNA using SuperScript II System (Invitrogen) and random hexamer primers. Pparγ1 and Pparγ2 gene expression was quantified by qRT-PCR using isoform-specific primers and SYBR Green chemistry on a LightCycler 480 (Roche, Penzberg, Germany).

### Statistical analyses

Statistical methods pertaining to the network and other associated analysis of gene expression and gene set data were as detailed above. Kaplan-Meier survival analysis, which allows for censored cases, was used to analyze the survival data in SPSS (version 14). Metabolic and molecular data for *Pparg^hyp/hyp^* and *Pparg2^−/−^* mice were analyzed using Student's t-test and are presented as means ± s.e.m.

## Supporting Information

Figure S1 *Pparg1* and *Pparg2* gene expression in WAT, BAT, liver and skeletal muscle in mouse models with altered *Pparg* locus. Data are presented relative to mean WAT expression in the wild type (*Pparg* ^+/+^) for each *Pparg* isoform. Note the much lower expression levels in liver and muscle. (A) Hypomorphic *Pparg* deficient mouse. (B) *Pparg2* knock-out mouse. Note that only one mouse per group was analyzed.
(0.07 MB TIF)

Figure S2 Metabolic phenotype of ∼2 year old *Pparg2* knock-out mice. For all tests, *n* = 4–9 per group. (A) Unaltered body weight and (B) fat content were analyzed by QNMR and are presented in % of fat of total body weight. (C) Intraperitoneal glucose tolerance test. The mean areas under the curve above baseline (AUC) are shown in the inset. (D) HOMA index for insulin resistance, calculated from fasting glucose and insulin values. (E) Fasting insulin and (F) adiponectin levels. None of the comparisons showed statistical significance.
(0.06 MB TIF)

Table S1 The seed set of 60 “known” longevity genes in mouse for the identification of a novel transcriptional longevity subnetwork. The gene list was derived from public resources (see Materials and Methods). The overlaps with the consensus white adipose tissue network (33 genes) and the rosiglitazone signature in the mouse WAT (5 genes) are indicated by a plus sign.
(0.03 MB XLS)

Table S2 Listing of 742 potentially novel longevity genes. Genes are ranked for the strength of connectivity with the network of the 33 “known” longevity genes within the male mouse adipose tissue transcriptional network of 13,088 genes.
(0.12 MB XLS)

Text S1 Supporting Methods.
(0.18 MB DOC)
