# Supplementary figures and images for: Pparγ2 Is a Key Driver of Longevity in the Mouse
Source: PLoS Genet. 2009 Dec 4;5(12):e1000752. doi: 10.1371/journal.pgen.1000752 (PMC2780700; doi:10.1371/journal.pgen.1000752)

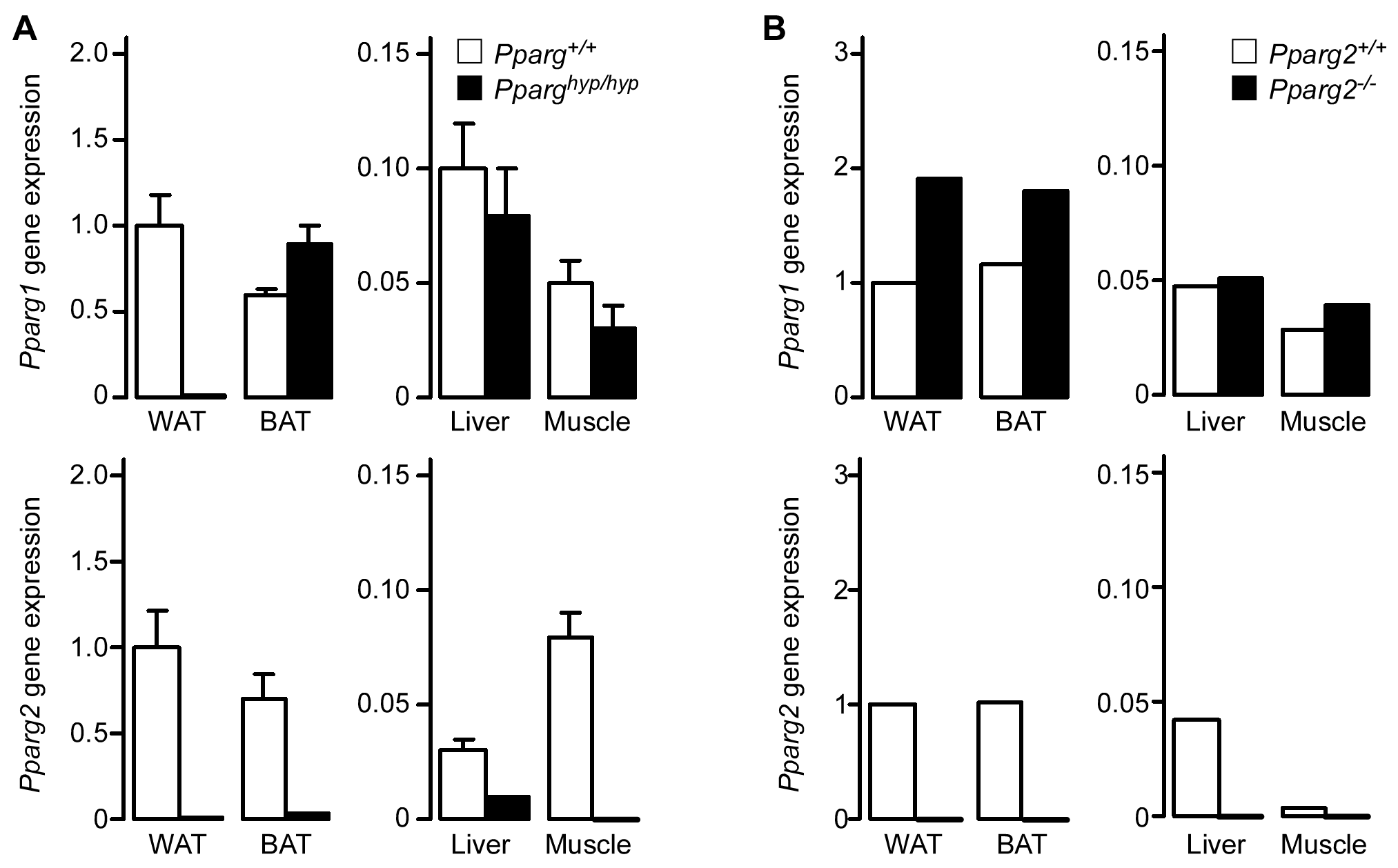

Supplement: Figure S1 — Pparg1 and Pparg2 gene expression in WAT, BAT, liver and skeletal muscle in mouse models with altered Pparg locus. Data are presented relative to mean WAT expression in the wild type (Pparg +/+) for each Pparg isoform. Note the much lower expression levels in liver and muscle. (A) Hypomorphic Pparg deficient mouse. (B) Pparg2 knock-out mouse. Note that only one mouse per group was analyzed. (0.07 MB TIF) [file pgen.1000752.s001.tif]

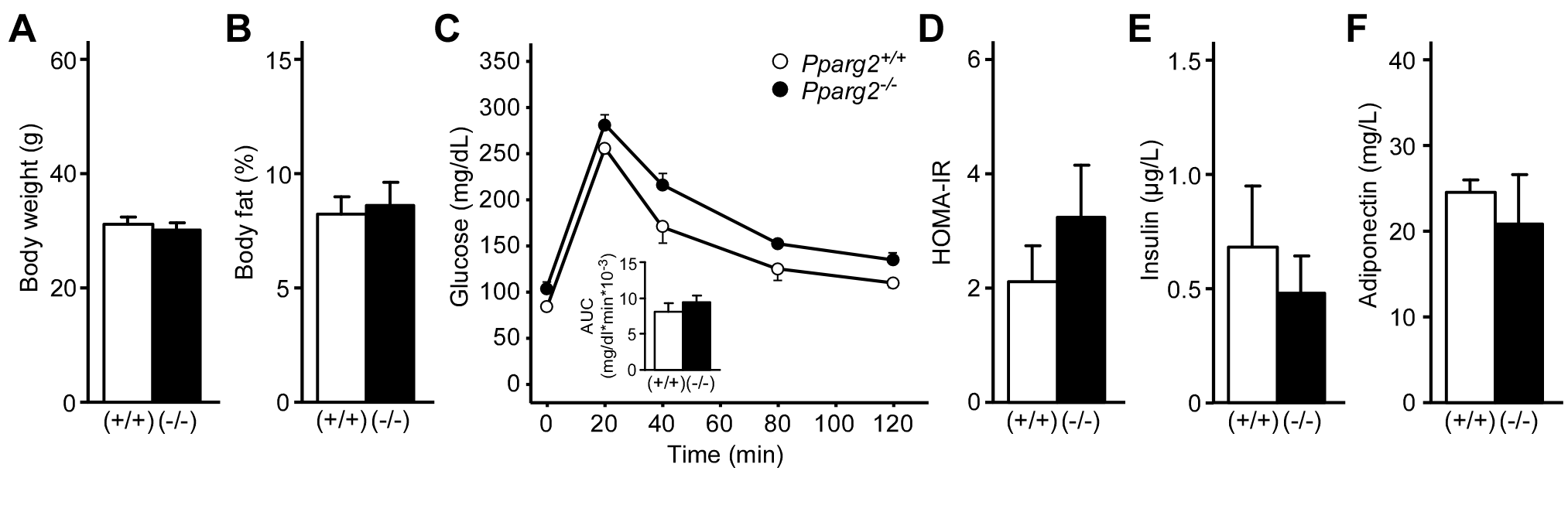

Supplement: Figure S2 — Metabolic phenotype of ∼2 year old Pparg2 knock-out mice. For all tests, n = 4–9 per group. (A) Unaltered body weight and (B) fat content were analyzed by QNMR and are presented in % of fat of total body weight. (C) Intraperitoneal glucose tolerance test. The mean areas under the curve above baseline (AUC) are shown in the inset. (D) HOMA index for insulin resistance, calculated from fasting glucose and insulin values. (E) Fasting insulin and (F) adiponectin levels. None of the comparisons showed statistical significance. (0.06 MB TIF) [file pgen.1000752.s002.tif]
