# Supplementary material for: Pparγ2 Is a Key Driver of Longevity in the Mouse
Source: PLoS Genet. 2009 Dec 4;5(12):e1000752. doi: 10.1371/journal.pgen.1000752 (PMC2780700; doi:10.1371/journal.pgen.1000752)
Supplement: Text S1 — Supporting Methods. (0.18 MB DOC) [file pgen.1000752.s005.doc]

**Supporting Information**

**Supporting Methods**

***Mouse F2 crosses.*** Four different mouse F2 crosses, BXH/ApoE^-/-^, BXH/wt, BXC and BXA, were used in this study. All mice were maintained according to the guidelines of the American Association for Accreditation of Laboratory Animal Care (AAALAC). Each cross is briefly described in the following:

The *BXH/ApoE*^–/–^ cross was previously described [1]. C57BL/6J ApoE null (B6.ApoE^–/–^) were purchased from Jackson Laboratory. C3H/HeJ ApoE null (C3H.Apo E^–/–^) were generated by backcrossing B6.ApoE^–/–^ to C3H for ten generations. F1 mice were generated from reciprocal intercrossing between B6.ApoE^–/–^ and C3H.ApoE^–/–^, and F2 mice were subsequently bred by intercrossing F1 mice. A total of 334 (169 female, 165 male) were bred, and all were fed Purina Chow containing 4% fat until 8 wk of age, and then transferred to western diet containing 42% fat and 0.15% cholesterol for 16 wk. Mice were killed at 24 wk, and liver, white adipose tissue, and whole brains were immediately collected and flash-frozen in liquid nitrogen.

The *BXH/wt* cross was describe previously [2]. C57BL/6J wild type mice and C3H/HeJ wild type mice were intercrossed to generate 309 F2 mice (164 males and 145 females). F2 mice were fed a chow diet containing 4% fat until 8 wk of age and were placed on a high-fat “Western” diet containing 42% fat and 0.15% cholesterol for 12 wk. All mice were maintained on a 12-h light/dark cycle. At 20 wk, mice were killed after a 12-h fast, and adipose tissue was dissected, flash frozen in liquid nitrogen, and stored at −80°C.

The *BXC* cross was described previously [3]. C57BL/6J (B6) mice were intercrossed with Castaneus (CAST) mice to generate 442 F2 progeny (276 females, 166 males). All mice were maintained on a 12 h light–12 h dark cycle and fed ad libitum. BXC mice were fed Purina Chow until 10 wk of age, and then fed western diet (Teklad 88137, Harlan Teklad) for the subsequent 8 wk. Mice were fasted overnight before they were killed. Their liver, adipose, muscle and brain tissues were collected, flash frozen in liquid nitrogen, and stored in −80 °C prior to RNA isolation.

The *BXA* cross was generated by intercrossing C57BL/6J (B6) and A/J mice. 359 F2 mice (180 females and 179 males) were fed a chow diet until 6 wk of age, then fed a high fat diet for 10 weeks. In week 16, mice were fasted for 4 hours prior to CO_2_ asphyxiation and necropsy. Their liver and adipose tissues were collected, flash frozen in liquid nitrogen, and stored in −80 °C prior to RNA isolation.

***Inferring causal relationships between expression traits****.* For two expression traits $T_{1}$ and $T_{2}$ linked to the same locus $L$ in the yeast cross, there are three basic relationships that are possible between the two traits relative to the DNA locus $L$. Either DNA variations at the locus $L$ lead to changes in trait $T_{1}$ that in turn lead to changes in trait $T_{2}$, or variations at locus $L$ lead to changes in trait $T_{2}$ that in turn lead to changes in trait $T_{1}$, or variations at locus $L$ independently lead to changes in traits $T_{1}$ and $T_{2}$, as previously described [4]. Assuming standard Markov properties for these basic relationships, the joint probability distributions corresponding to these 3 models, respectively, are:

$$P\left(L,T_{1},T_{2}\right)=P\left(L\right)P\left(T_{1}|L\right)P\left(T_{2}|T_{1}\right)$$

$$P\left(L,T_{1},T_{2}\right)=P\left(L\right)P\left(T_{2}|L\right)P\left(T_{1}|T_{2}\right)$$

$P\left(L,T_{1},T_{2}\right)=P\left(L\right)P\left(T_{2}|L\right)P\left(T_{1}|T_{2},L\right)$,

where the final term on the right-hand side of equation M3 reflects that the correlation between $T_{1}$ and $T_{2}$ may be explained by other shared loci or common environmental influences, in addition to locus $L$. We assume Markov equivalence between $T_{1}$ and $T_{2}$ for model M3 so that $P\left(T_{2}|L\right)P\left(T_{1}|T_{2},L\right)=P\left(T_{1}|L\right)P\left(T_{2}|T_{1},L\right)$. $P\left(L\right)$ is the genotype probability distribution for locus $L$ and is based on a previously described recombination model [5]. The random variables $T_{1}$ and $T_{2}$ are taken to be normally distributed about each genotypic mean at the common locus $L$, so that the likelihoods corresponding to each of the joint probability distributions are then based on the normal probability density function, with mean and variance for each component given by: 1) for $P\left(T_{1}|L\right)$ the mean and variance are and $Var\left(T_{1}|L\right)=\sigma_{T_{1}}^{2}$, 2) for $P\left(T_{2}|L\right)$ the mean and variance are and $Var\left(T_{2}|L\right)=\sigma_{T_{2}}^{2}$, and 3) for $\text{ }P\left(T_{1}|T_{2}\right)$ the mean and variance are $E\left(T_{1}|T_{2}\right)=\mu_{T_{1}}+\rho\frac{\sigma_{T_{1}}}{\sigma_{T_{2}}}\left(T_{2}-\mu_{T_{2}}\right)$ and $Var\left(T_{1}|T_{2}\right)=\left(1-\rho^{2}\right)\sigma_{_{T_{1}}}^{2}$, where $\rho$ represents the correlation between $T_{1}$ and $T_{2}$, and and are the genotypic specific means for $T_{1}$ and $T_{2}$, respectively. The mean and variance for $P\left(T_{2}|T_{1}\right)$ follow similarly from that given for $P\left(T_{1}|T_{2}\right)$. From these component pieces, the likelihoods for each model are formed by multiplying the densities for each of the component pieces across all of the individuals in the population [4]. The likelihoods are then compared among the different models in order to infer the most likely of the three. Because the number of model parameters among the models differs, a penalized function of the likelihood was used to avoid the bias against parsimony. The model with the smallest value of the penalized statistic $-2\log\text{ }\mathrm{L}_{i}(\hat{\mathbf{\theta}}_{i}|L,R,C)+k\times p_{i}$ was chosen. Here, $\mathrm{L}_{i}(\hat{\mathbf{\theta}}_{i}|L,R,C)$is the maximum likelihood for the *i*th model, *p_i_* is the number of parameters in the *i*th model, and *k* is a constant. In this instance we took the penalized statistic to be the Bayesian Information Criteria (BIC) where *k* is set to $\log\left(n\right)$, with *n* denoting the number of observations.

***Incorporating genetic data as a network prior in the Bayesian network reconstruction process****.* In general, Bayesian networks can only be solved to Markov equivalent structures, so that it is often not possible to determine the direction of a given edge. However, the Bayesian network reconstruction algorithm can take advantage of the experimental cross design (or segregating populations more generally) by incorporating genetic data to break the symmetry among nodes in the network that lead to Markov equivalent structures, thereby providing a way to direct edges in the network in an unambiguous fashion [6]. We modified the reconstruction algorithm to incorporate genetic data as prior evidence that two genes may be causally related, as previously described [6]. The genetic priors are constructed from three basic sources: (I) Genes with cis eQTLs [7] are allowed to be parent nodes of genes with coincident trans eQTLs, $p(cis->trans)=1$, but genes with trans eQTLs are not allowed to be parents of genes with cis eQTLs, $p(trans->cis)=0$. (II) The eQTL analysis described above was carried out to identify suggestive eQTLs for all expression traits [8] (LOD scores greater than 2.8, corresponding to a point-wise p-value of 0.0003 in the present setting, less than 1 QTL expected by random across genome). Genes from this analysis with cis or trans eQTL were then tested individually for pleiotropic effects at each of their eQTL to determine whether any other genes in the set were driven by the same eQTL [5,9]. If such pleiotropic effects were detected, the corresponding gene pair and locus giving rise to the pleiotropic effect were used to infer a causal/reactive or independent relationship based on a formal causality test described above [4]. The reliabilities of the inferred relationship between genes A and B at a given locus $l_{i}$, $p(A->B|A,B,l_{i})$, $p(B->A|A,B,l_{i})$, and $p(A \perp B|A,B,l_{i})$, were then estimated by a standard bootstrapping procedure [10]. If an independent relationship is inferred ($p(A \perp B|A,B,l_{i})>0.5$), then the prior probability that gene A is a parent of gene B is scaled as$p(A->B)=1-\frac{\sum_{i} p(A\perp B|A,B,l_{i})}{\sum_{i} 1}$, where the sums are taken over all loci used to infer the relationship. If a causal or reactive relationship is inferred ($p(A->B|A,B,l_{i})$ or $p(B->A|A,B,l_{i})$ is greater than 0.5) then the prior probability is scaled as $p(A->B)=\frac{2*\sum_{i} p(A->B|A,B,l_{i})}{\sum_{i} p(A->B|A,B,l_{i})+p(B->A|A,B,l_{i})}$. (III) If the causal/reactive relationship between genes A and B could not be determined by sources (I) or (II), then the complexity of the eQTL signature for each gene was taken into consideration. Genes with a simpler, albeit stronger eQTL signature (i.e., a small number of eQTL that explain the genetic variance component for the gene, with a significant proportion of the overall variance explained by the genetic effects) were taken to be more likely to be causal compared to genes with more complex and possibly weaker eQTL signatures (i.e., a larger number of eQTL explaining the genetic variance component for the gene, with less of the overall variance explained by the genetic effects). The structure prior that gene A is a parent of gene B was then taken to be $p(A->B)=2*\frac{1+n(B)}{2+n(A)+n(B)}$ , where n(A) and n(B) are the number of eQTLs with LOD scores greater than 2.8 for A and B, respectively. We have found that both information on cis-acting eQTLs (excluding edges into certain nodes) and information on trans-acting eQTLs (increasing the likelihood of some edges over others) improve the quality of the network reconstruction [11].

***Generation of the transcriptional network for mouse adipose tissue.*** We obtained male adipose tissue gene expression data from the four mouse F2 crosses described above using Agilent microarrays, and generated a Bayesian network for each cross by integrating genetic and gene expression data [12-14]. For each cross, one thousand Bayesian networks were reconstructed using different random seeds to start the reconstruction process. From the resulting set of 1000 networks generated by this process, edges that appeared in greater than 30% of the networks were used to define a consensus network. Edges in this consensus network were removed if 1) the edge was involved in a loop, and 2) the edge was the most weakly supported of all edges making up the loop. There are 5269, 8727, 6698 and 6029 nodes, 1603, 7831, 6851 and 7085 links for the BXH/ApoE^-/-^, BXH/wt, BXC and BXA cross networks, respectively. The final adipose transcriptional network, containing 13088 nodes and 22809 edges, was obtained as the union of all these 4 separate Bayesian networks.

**References**

1. Wang S, Yehya N, Schadt EE, Wang H, Drake TA, et al. (2006) Genetic and Genomic Analysis of a Fat Mass Trait with Complex Inheritance Reveals Marked Sex Specificity. PLoS Genet 2: e15.

2. Farber CR, van Nas A, Ghazalpour A, Aten JE, Doss S, et al. (2009) An integrative genetics approach to identify candidate genes regulating BMD: combining linkage, gene expression, and association. J Bone Miner Res 24: 105-116.

3. Schadt EE, Molony C, Chudin E, Hao K, Yang X, et al. (2008) Mapping the genetic architecture of gene expression in human liver. PLoS Biol 6: e107.

4. Schadt EE, Lamb J, Yang X, Zhu J, Edwards S, et al. (2005) An integrative genomics approach to infer causal associations between gene expression and disease. Nat Genet 37: 710-717.

5. Jiang C, Zeng ZB (1995) Multiple trait analysis of genetic mapping for quantitative trait loci. Genetics 140: 1111-1127.

6. Zhu J, Lum PY, Lamb J, GuhaThakurta D, Edwards SW, et al. (2004) An integrative genomics approach to the reconstruction of gene networks in segregating populations. Cytogenet Genome Res 105: 363-374.

7. Doss S, Schadt EE, Drake TA, Lusis AJ (2005) Cis-acting expression quantitative trait loci in mice. Genome Res 15: 681-691.

8. Kruglyak L, Lander ES (1995) A nonparametric approach for mapping quantitative trait loci. Genetics 139: 1421-1428.

9. Lum PY, Chen Y, Zhu J, Lamb J, Melmed S, et al. (2006) Elucidating the murine brain transcriptional network in a segregating mouse population to identify core functional modules for obesity and diabetes. J Neurochem 97 Suppl 1: 50-62.

10. Sieberts SK, Schadt EE (2007) Handbook of Statistical Genetics; (Editor) DJB, (Co-Editor) CC, (Co-Editor) MB, editors.

11. Zhu J, Wiener MC, Zhang C, Fridman A, Minch E, et al. (2007) Increasing the Power to Detect Causal Associations by Combining Genotypic and Expression Data in Segregating Populations. PLoS Comput Biol 3: e69.

12. Zhu J, Wiener MC, Zhang C, Fridman A, Minch E, et al. (2007) Increasing the power to detect causal associations by combining genotypic and expression data in segregating populations. PLoS Comput Biol 3: e69.

13. Zhu J, Lum PY, Lamb J, GuhaThakurta D, Edwards SW, et al. (2004) An integrative genomics approach to the reconstruction of gene networks in segregating populations. Cytogenet Genome Res 105: 363-374.

14. Schadt EE, Lamb J, Yang X, Zhu J, Edwards S, et al. (2005) An integrative genomics approach to infer causal associations between gene expression and disease. Nat Genet 37: 710-717.
